# *CRTC1* gene is differentially methylated in the human hippocampus in Alzheimer’s disease

**DOI:** 10.1186/s13195-016-0183-0

**Published:** 2016-04-19

**Authors:** Maite Mendioroz, Naiara Celarain, Miren Altuna, Javier Sánchez-Ruiz de Gordoa, María Victoria Zelaya, Miren Roldán, Idoya Rubio, Rosa Larumbe, María Elena Erro, Iván Méndez, Carmen Echávarri

**Affiliations:** NeuroEpigenetics Laboratory, Navarrabiomed- IdiSNA (Navarra Institute for Health Research), c/ Irunlarrea, 3, Pamplona, Navarra 31008 Spain; Department of Neurology, Complejo Hospitalario de Navarra- IdiSNA (Navarra Institute for Health Research), Pamplona, Navarra 31008, Spain; Department of Pathology, Complejo Hospitalario de Navarra- IdiSNA (Navarra Institute for Health Research), Pamplona, Navarra 31008 Spain; Hospital García-Orcoyen, Estella, Navarra 31200 Spain; Hospital Psicogeriátrico Josefina Arregui, Alsasua, Navarra 31800 Spain

**Keywords:** *CRTC1*, Epigenetics, DNA methylation, Alzheimer’s disease, Hippocampus

## Abstract

**Background:**

*CRTC1* (*CREB* regulated transcription coactivator 1) gene plays a role in synaptic plasticity, learning and long-term memory formation in the hippocampus. Recently, *CRTC1* has been shown to be downregulated in Alzheimer’s disease (AD). Nevertheless, the mechanisms underlying *CRTC1* dysregulation in AD remain unclear.

**Methods:**

To understand better the epigenetic mechanisms regulating *CRTC1* expression that may be altered in AD, we profiled DNA methylation at CpG site resolution by bisulfite cloning sequencing in two promoter regions (referred to as Prom1 and Prom2) of the *CRTC1* gene in human hippocampus from controls and AD cases. Next, we correlated DNA methylation levels with AD-related pathology, i.e., β-amyloid and phosphorylated-tau (p-tau) burden and also measured *CRTC1* mRNA levels by RT-qPCR.

**Results:**

Methylation levels were lower in AD cases as compared to controls within both promoter regions (Prom1: 0.95 % vs. 5 %, *p*-value < 0.01 and Prom2: 2.80 % vs. 17.80 %, *p*-value < 0.001). Interestingly, *CRTC1* methylation levels inversely correlated with AD-related neuropathological changes, particularly with p-tau deposition (r_Spearman_ = -0.903, *p* < 0.001). Moreover, a 1.54-fold decrease in *CRTC1* mRNA levels was observed in hippocampus of AD cases compared to controls (*p* < 0.05) supporting the notion that *CRTC1* is downregulated in the AD hippocampus.

**Conclusions:**

DNA methylation levels within two distinct promoter regions of the *CRTC1* gene were decreased in human hippocampus affected by AD compared with controls and methylation within Prom1 showed a strong inverse correlation with p-tau deposition. Further studies are guaranteed to elucidate the precise role that *CRTC1* methylation plays in AD pathophysiology.

**Electronic supplementary material:**

The online version of this article (doi:10.1186/s13195-016-0183-0) contains supplementary material, which is available to authorized users.

## Background

*CRTC1* (*CREB* regulated transcription coactivator 1) [EMBL: BC017075] plays a critical role as coactivator of the *CREB* (cAMP-responsive element binding)-dependent gene transcription pathway [1], which in turn is essential to synaptic plasticity, learning and long-term memory formation in the hippocampus [2–7]. *CRTC1* is highly expressed in brain, particularly in hippocampal neurons [8, 9], where it translocates from synapses and dendrites to the nucleus upon the concomitant activation of calcium and cAMP signaling pathways [6, 10, 11]. Moreover, it has been shown that *CRTC1* is involved in dendritic growth of developing cortical neurons [12]. The relevance of this gene to brain function is additionally supported by experiments performed on knockout mouse models. Mice lacking *Crtc1* develop psychomotor retardation along with neurobehavioral endophenotypes. Interestingly, these mice show reduced dopamine and serotonin turnover in the prefrontal cortex and decreased expression of several downstream genes involved in neuroplasticity [13]. In addition, *CRCT1* was recently identified as a modulator of mitochondrial metabolism and longevity [14, 15].

All this evidence makes *CRTC1* an intriguing candidate to be examined in neurodegenerative disorders at the molecular level [16]. Indeed, the role of *CRCT1* in Alzheimer’s disease (AD) has been previously studied in mouse models. Gene transcription mediated by *CRTC1* is impaired in neurons and brain from APP (Sw, Ind) transgenic mice, an AD mouse model overexpressing the human β-amyloid precursor protein (APP) [PDB: E9PG40] carrying human mutations [17, 18]. Remarkably, β-amyloid-induced spatial learning and memory deficits in APP (Sw, Ind) transgenic mice are reversed by restoring a specific subset of *CRTC1* target genes [19]. However, studies performed on postmortem human brain samples are still very scarce. In this regard, a recent report shows that *CRTC1*-dependent genes and CRTC1 protein [PDB: Q6UUV9] levels are significantly reduced in human hippocampus at intermediate and advanced (IV-VI) Braak pathological stages [19]. Nevertheless, the specific mechanisms underlying *CRTC1*-dependent gene transcriptional disruption in AD still remain unclear.

DNA methylation is a widely studied epigenetic mechanism that contributes to regulate gene expression. The addition of a methyl group to the 5-carbon position of the cytosine-phosphate-guanine (CpG) sites, mostly located at regulatory elements in the genome, modulates the transcription of nearby or related genes. Interestingly, DNA methylation patterns change during the ageing process [20–22] and may have an impact on gene expression in the human brain [23]. In addition, aberrant DNA methylation patterns are involved in an increasing number of common diseases [24, 25], including neurodegenerative conditions. Based on that evidence, we hypothesized that certain regulatory elements of the *CRTC1* gene may be differentially methylated in the hippocampus of AD patients. Hence, we aimed to profile DNA methylation levels by bisulfite cloning sequencing in two distinct promoter regions of *CRTC1* in hippocampal samples from a cohort of neuropathologically defined “pure” AD cases and controls. We also explored the relationship between DNA methylation levels and AD-related neuropathological changes. Next, *CRTC1* mRNA expression was assessed in AD and control hippocampus.

## Methods

### Human brain samples

We conducted an observational, case-control study comparing postmortem hippocampal samples from AD patients and control hippocampus. Frozen postmortem hippocampal samples from 30 Alzheimer’s disease (AD) cases and 12 controls were provided by the Navarrabiomed Brain Bank. After death, half brain specimens from donors were cryopreserved at −80 °C.

### Immunostaining protocol

Formalin-fixed (4 %), paraffin-embedded tissue from mid-hippocampus region was cut (5-mm sections) and placed on StarFrost Microscope Slides. After deparaffinizing, endogenous peroxidase activity was quenched by incubation with 0.3 % (vol/vol) H2O2 in methanol for 30 minutes at room temperature. Antigen retrieval was performed by soaking the sections in 10-mM citrate buffer pH 6.0, heated and boiled for 10 minutes in a microwave oven.

Assessment of β-amyloid deposition was carried out with a mouse monoclonal (S6F/3D) anti β-amyloid antibody (Leica Biosystems Newcastle Ltd, Newcastle upon Tyne, UK) which identified the presence of amyloid pathology.

Evaluation of neurofibrillary pathology was performed with a mouse monoclonal antibody anti-human PHF-TAU, clone AT-8, (Tau AT8) (Innogenetics, Ghent, Belgium), which identified hyperphosphorylated tau (p-tau) [26] in the shape of dystrophic neurites and neurofibrillary tangles. The reaction product was visualized using an automated slide immunostainer (Leica Bond Max) with Bond Polymer Refine Detection (Leica Biosystems, Newcastle Ltd). Omission of the primary antibody was used as a negative control for all stainings.

### Neuropathological assessment

Neuropathological examination was completed following the usual recommendations [27] and assessment of AD was performed according to the updated National Institute on Aging-Alzheimer's Association guidelines [26]. Samples were further classified, based on the ABC score, into four groups: control (*n* = 12), initial AD (including A1B1C1, A1B2C1, A1B2C2, and A1B2C3 scores) (*n* = 9), intermediate AD (including A2B2C2, A2B2C3, and A2B3C3 scores) (*n* = 7) and advanced AD (including A3B2C1, A3B2C3, A3B3C2, and A3B3C3 scores) (*n* = 14) (Additional file 1: Figure S1). Importantly, to avoid spurious associations, those individuals showing coexisting protein deposits different from p-tau or β-amyloid were not eligible for the study. This approach maximizes chances of finding true associations with AD, even though reducing the final sample size. Neuropathological and demographic features of subjects, including age, gender, ABC score and postmortem interval are listed in Additional file 2: Table S1.

### Quantitative assessment of β-amyloid and p-tau deposits in brain tissues

In order to quantitatively assess the β-amyloid and p-tau burden for further statistical analysis we applied a method to quantify protein deposits. This method generates a numeric measurement that reflects the extent of β-amyloid and p-tau deposition. Sections of the mid hippocampus were examined after performing immunostaining with anti β-amyloid and anti p-tau antibodies as described above. Three pictures were obtained for each immunostained section using an Olympus BX51 microscope at 10x magnification power. Morphological deposits of β-amyloid, as described by Braak and Braak (neuritic, immature and compact plaque), were manually determined and those areas were further edited and analyzed with ImageJ software [28] (Additional file 1: Figure S2). Next, β-amyloid plaque count referred to as amyloid plaque score (APS) and total area of β-amyloid deposition were automatically measured by ImageJ and averaged for each section. Regarding p-tau deposit, pictures were also analyzed with ImageJ software, by adjusting in the threshold color hue (maximum range: 45–197), saturation (maximum range: 7–243) and brightness (maximum range: 50–195), in order to obtain an averaged quantitative measure of the global p-tau deposit for each section (Additional file 1: Figure S2).

### *CRTC1 m*ethylation profiling by bisulfite cloning sequencing

Genomic DNA was isolated from hippocampal tissue by standard methods [29]. Next, 500 ng of genomic DNA was bisulfite converted using the EpiTect Bisulfite Kit (QIAGEN, Redwood City, CA, USA) according to the manufacturer’s instructions. Two promoter regions (Prom1 and Prom2) within the *CRTC1* gene were amplified by PCR (Additional file 1: Figure S3). Genomic coordinates were obtained from GRCh37/Hg19 assembly. Primer pair sequences were designed by MethPrimer [30] and are listed in Additional file 2: Table S2. PCR products were cloned using the TopoTA Cloning System (Invitrogen, Carlsbad, CA, USA) and a minimum of 12 independent clones were sequenced for each examined subject and region. Methylation graphs were obtained with the QUMA software [31].

### *CRTC1* mRNA expression analysis

Total RNA was isolated from the 42 hippocampus homogenates using RNeasy Lipid Tissue Mini kit (QIAGEN, Redwood City, CA, USA), following the manufacturer’s instructions. Genomic DNA was removed with recombinant DNase (TURBO DNA-free™ Kit, Ambion, Inc., Austin, TX, USA). RNA integrity was checked by 1.25 % agarose gel electrophoresis under denaturing conditions. Concentration and purity of RNA were both evaluated with a NanoDrop spectrophotometer. Only RNA samples showing a minimum quality index (260 nm/280 nm absorbance ratios between 1.8 and 2.2 and 260 nm/230 nm absorbance ratios higher than 1.8) were included in the study. Complementary DNA (cDNA) was reverse transcribed from 1500 ng total RNA with SuperScript® III First-Strand Synthesis Reverse Transcriptase (Invitrogen, Carlsbad, CA, USA) after priming with oligo-d (T) and random primers. RT-qPCR reactions were performed in triplicate with Power SYBR Green PCR Master Mix (Invitrogen, Carlsbad, CA, USA) in an Applied 7300 Real-Time PCR System (Applied Biosystems, Foster City, CA, USA). Sequences of primer pair were designed using the Real Time PCR tool (IDT, Coralville, IA, USA) and are listed in Additional file 2: Table S2. Relative expression level of *CRTC1* mRNA in a particular sample was calculated as previously described [32] and the geometric mean of *GAPDH* and *ACTB* genes were used to normalize expression values.

### Statistical data analysis

Statistical analysis was performed with SPSS 21.0 (IBM, Inc., USA). Data represent the mean ± SEM or median (range), depending on the type of variable. Differences with *p*-value < 0.05 were considered significant. Statistical significance for expression and bisulfite intergroup differences were assessed by the Mann-Whitney U test and the Kruskal–Wallis test. Spearman’s rank correlation coefficient was used to determine correlation between AD-related pathology and methylation levels. When multiple comparisons were performed, as was the case of correlation between methylation levels and AD-related pathological burden, a Bonferroni correction was applied and the significance threshold was set at p-corrected value = 0.008. GraphPad Prism version 6.00 for Windows (GraphPad Software, La Jolla, CA, USA) was used to draw the graphs except for methylation figures that were obtained by QUMA software.

### Ethics, consent and permissions

Our study was carried out in accordance with the Declaration of Helsinki and handling of human brain samples was performed according to the current Spanish national legislation (Law 14/2007 and Royal Decree RD1716/2011). The Ethics Committee of the “Complejo Hospitalario de Navarra” approved the use of human subjects for this study (90/2014). Written informed consent was obtained from all subjects or next of kin, previous to brain donation, to perform research projects related to neurodegenerative conditions. The consent form is held by the authors’ institution and is available for review by the Editor-in-Chief.

## Results

### *CRTC1* methylation is decreased in hippocampus of AD cases compared to controls

**Fig. 1:**
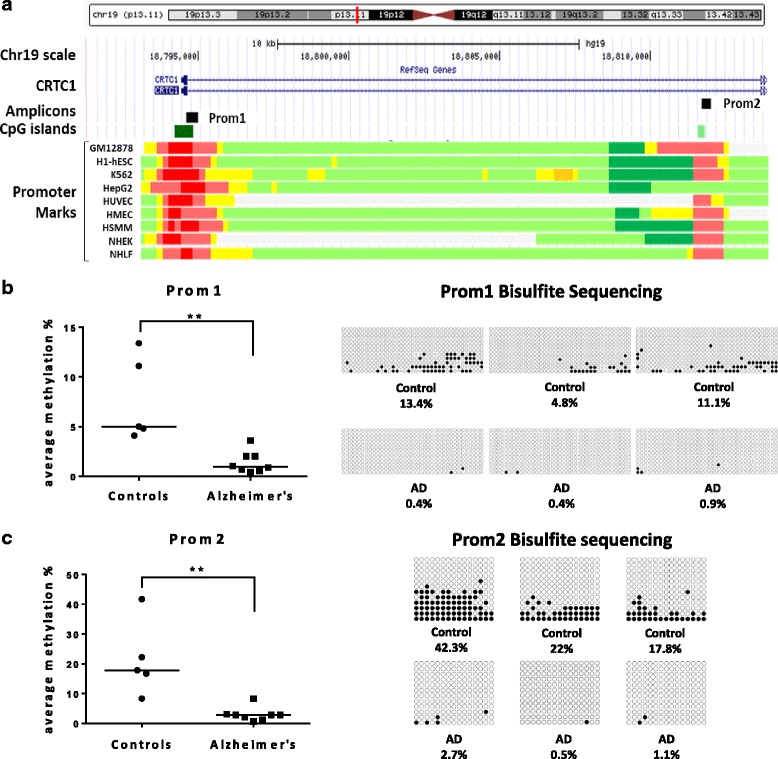
DNA methylation levels within two promoter regions of *CRTC1* in Alzheimer’s disease (AD) and control hippocampus. **a** The graph shows genomic position of the two amplicons (Prom1 and Prom2) within the two promoter regions of the *CRTC1* gene that were examined by bisulfite cloning sequencing. At the *bottom* of the graph, predicted functional elements are shown for each of nine human cell lines explored by chromatin immunoprecipitation (ChIP) combined with massively parallel DNA sequencing. *Boxes* represent promoter regions (red), enhancers (yellow), transcriptional transition and elongation (dark green) and weak transcribed regions (light green). CpG islands are also represented by green boxes. The track was obtained from *Chromatin State Segmentation by HMM from ENCODE/Broad* track shown at the UCSC Genome Browser. Next, dot plot charts and representative examples of the methylation graphs for Prom1 (**b**) and Prom2 (**c**), respectively, are shown. A decrease in methylation was observed within both promoters in AD hippocampus compared to controls. *Horizontal lines* within dot plots represent median methylation values for each group. *Boxes on the right* represent individual patients. Black and white *circles* denote methylated and unmethylated cytosines, respectively. Each column symbolizes a unique CpG site in the examined amplicon and each line represents an individual DNA clone. *p-value < 0.05; ** *p*-value < 0.005. *CpG* cytosine-phosphate-guanine

To begin to ask whether DNA methylation within *CRTC1* is altered in AD hippocampus, two different promoters of the *CRTC1* gene were examined by bisulfite cloning sequencing. Promoters were identified by using chromatin immunoprecipitation sequencing (ChIP-seq) data from the track *Chromatin State Segmentation by HMM (Hidden Markov Model) from ENCODE project and Broad Institute,* shown at the UCSC Genome Browser [33]. This track displays chromatin state segmentation data for each of nine human cell types (Fig. 1a, Additional file 1: Figure S3). We designed primers to amplify two independent amplicons. Promoter 1 (Prom1) amplicon was placed at the 5’ end of *CRTC1*, partially overlapping exon 1 and the beginning of intron 1, and contained 37 CpG sites within the southern shore of a 628 bp-CpG island (*chr19:18794192–18794819*). Whereas promoter 2 (Prom2) amplicon was located within intron 1 and encompassed 15 CpG sites at the southern shore of a 230 bp-CpG island (*chr19:18811562–18811791*) (Fig. 1a, Additional file 1: Figure S3). To sum up, a total of 52 individual CpG sites, representing 63.8 % and 88.2 % of each CpG island, respectively, were analyzed in a subset of five control and eight AD cases. To calculate the average methylation level, DNA methylation percentage was measured at CpG site resolution and further averaged across all the CpG sites for each promoter region and subject. Interestingly, we found that average methylation level at Prom1 was decreased in AD cases compared to controls (0.95 % vs. 5 %, *p*-value < 0.01) (Fig. 1b). Similarly, average methylation level was significantly decreased within Prom2 in AD cases compared to controls (2.80 % vs. 17.80 %, *p*-value < 0.001) (Fig. 1c).

### *CRTC1* methylation correlates with p-tau burden

Next, we aimed to correlate DNA methylation levels with AD-related neuropathological changes recorded from the hippocampus sections. In brief, p-tau and β-amyloid burden were measured and averaged by using the ImageJ software (Additional file 1: Figure S2). The amyloid plaque score (APS) was also recorded (Additional file 2: Table S1). Notably, the average methylation level within Prom 1 showed a strong inverse correlation with p-tau deposition (r_Spearman_ = −0.903, *p* < 0.001). Although we found a correlation between Prom1 average methylation level and β-amyloid burden (Table 1), the *p*-value for this correlation did not reach the Bonferroni-corrected significance threshold (corrected *p*-value = 0.008). Likewise, average methylation level within Prom2 was no longer correlated with p-tau deposition and β-amyloid burden after applying the Bonferroni correction (Table 1).

**Table 1:** Correlation tests between *CRTC1* methylation levels and Alzheimer's disease (AD)-related pathological changes

AD-related changes	Prom1 methylation level		Prom2 methylation	
	Spearman coefficient	raw *p*-value	Spearman coefficient	raw *p*-value
p-tau area	−0.903	0.000024^a^	−0.753	0.002
averaged β-amyloid area	−0.761	0.003	−0.651	0.012
APS	−0.761	0.003	−0.640	0.014

*p-tau* hyperphosphorylated tau; *APS* amyloid plaque score

^a^denotes those comparisons below the Bonferroni-corrected *p*-value = 0.008

### *CRTC1* mRNA levels are downregulated in Alzheimer’s disease hippocampus

**Fig. 2:**
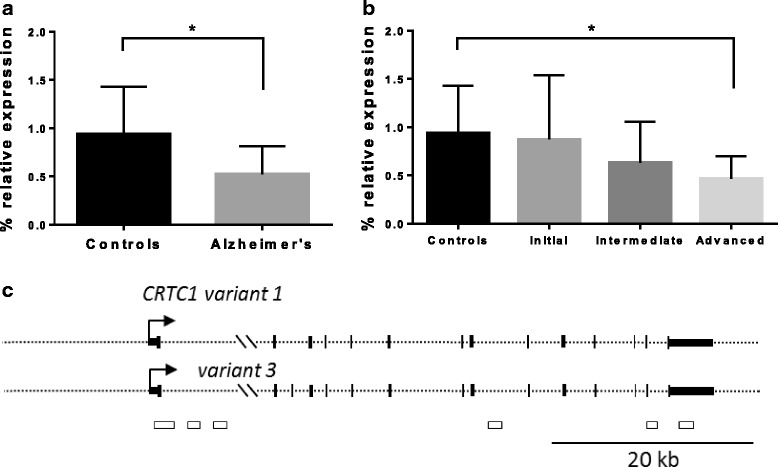
*CRTC1* mRNA expression is decreased in human hippocampus in Alzheimer’s disease (AD). **a** The graph shows a significant 1.54-fold decrease in *CRTC1* mRNA levels in AD hippocampal samples compared to control hippocampal samples. **b** *CRTC1* mRNA expression decreases across AD stages, as shown when *CRTC1* expression levels are sorted based on ABC score. *Boxes* represent percentage of *CRTC1* expression relative to the geometric mean of *GAPDH* and *ACTB* housekeeping genes expression. *Bars* represent the standard error of the mean. *p-value < 0.05; ** *p*-value < 0.005. **c** Two different *CRTC1* transcript variants are shown. *CRTC1* variant 1 has 14 exons and *CRTC1* variant 3 shares all the exons of variant 1 plus an additional exon 3. *Black boxes* represent exons, *arrows* represent transcription start sites and *white boxes* denote CpG islands. *CpG* cytosine-phosphate-guanine

To test if *CRTC1* was differentially expressed in our cohort, we measured *CRTC1* mRNA expression levels by RT-qPCR in hippocampal samples from Alzheimer’s disease (AD) cases and controls. Four samples did not pass the RNA quality threshold and so were not included in the experiments (see *CRTC1* mRNA expression analysis in the Methods section). Eventually, 26 AD cases were compared to 12 controls. RT-qPCR reactions were performed in triplicate for each sample and repeated twice within independent cDNA sets. Moreover, two different sets of primers (*CRTC1*-qPCR1 and *CRTC1*-qPCR2) were used to increase the reliability of the experiment (Additional file 2: Table S2). A 1.54-fold decrease in *CRTC1* mRNA levels was observed in the hippocampus of AD cases compared to controls (*p* < 0.05) (Fig. 2a). Next, a disease-staging analysis was conducted to investigate changes of *CRTC1* mRNA levels across AD stages. We found that *CRTC1* mRNA levels significantly decreased across AD stages (*p* < 0.05) and post-hoc analysis showed that *CRTC1* expression was significantly reduced at advanced stages compared to controls (*p* < 0.05) (Fig. 2b).

We further tested if differences in *CRTC1* mRNA expression resulted from a decrease in a particular type of *CRTC1* transcript variant. Two different *CRTC1* transcripts were identified in the Reference Sequence (RefSeq) database, i.e., *CRTC1* transcript variant 1 (NM_015321) and transcript variant 3 (NM_001098482), sharing all the exons but for an additional exon (exon 3) belonging to *CRTC1* variant 3 (Fig. 2c). Due to the extra exon 3, we were able to design a primer pair to selectively amplify *CRTC1* variant 3 (Additional file 2: Table S2), whereas a primer pair to amplify only *CRTC1* variant 1 was not able to be designed since all the exons of this variant are shared with *CRTC1* variant 3. We found that levels of *CRTC1* variant 3 mRNA were not different between AD cases and controls (*p* = 0.206), suggesting that significant differences in global *CRTC1* mRNA levels between AD cases and controls might result from a reduction in *CRTC1* variant 1 mRNA.

Finally, we studied the relationship between promoter methylation and mRNA expression for the *CRTC1* gene. We tested the correlation between DNA methylation levels and mRNA expression levels. In order to avoid potential bias from disease status, we tested the potential correlation separately in two groups, controls and AD patients. Interestingly, *CRTC1* mRNA expression showed a trend to be inversely correlated with Prom1 methylation levels (r_Spearman_ = −0.949, *p* = 0.051) in the control group; meanwhile, no significant correlation was found between methylation and expression among AD patients (r_Spearman_ = −0.287, *p* = 0.490).

### *mRNA levels of CRTC1 downstream target genes* are altered in Alzheimer’s disease hippocampus

We also wanted to test whether *CRTC1*-dependent genes were also dysregulated in our set of AD hippocampal samples. To this end, we measured mRNA levels of *BDNF*, c-fos, *EGR4*, and *EGR1* by RT-qPCR in our set of samples. Primer pairs and specific transcripts that were amplified by RT-qPCR are shown in Additional file 2: Table S2.

Interestingly, we found significantly decreased mRNA levels of *BDNF*, *EGR4*, and *EGR1* in AD hippocampus compared to control hippocampus (*p*-value < 0.05) (Additional file 1: Figure S4). These results are in accordance with previous reports based on AD in vitro models, murine models and human brain. On the contrary, c-fos mRNA expression was significantly increased in AD cases compared to controls in our set of samples (*p*-value < 0.005) (Additional file 1: Figure S4).

## Discussion

Here we report altered patterns of DNA methylation within two distinct promoter regions of *CRTC1* in the human hippocampus affected by Alzheimer’s disease (AD). Interestingly, DNA methylation levels are decreased in AD and inversely correlate with p-tau burden in the hippocampus.

Our results indicate that important regulatory regions for the *CRTC1* gene are differentially methylated in the AD hippocampus compared to controls. It is well known that *CRTC1* is mainly expressed in the CA1 region of the hippocampus [8] and is essential to synaptic plasticity and memory consolidation [2–7]. Moreover, studies conducted on AD mouse models highlight its relevance to AD pathogenesis [3, 17–19] and CRTC1 protein has been found to be downregulated in the human hippocampus affected by AD [19]. Thus, studying regulatory mechanisms of *CRTC1* gene expression in the human hippocampus, one of the more vulnerable regions to AD pathology, is of utmost interest. Our finding suggests that altered patterns of methylation at regulatory regions might contribute to disruption of the *CRTC1* gene function that was previously described in AD [19], as DNA methylation is one of the most important epigenetic mechanisms that regulate gene expression. Most interestingly, methylation levels correlated to AD-related pathology changes in our cohort. In particular, a strong inverse correlation was found between DNA methylation levels and p-tau burden in the hippocampus (r_Spearman_ = −0.903, *p* < 0.001). It is of note that neurofibrillary tangles, which are aggregates of p-tau protein that accumulate within neurons, are found in the first stages of AD restricted to the hippocampus and entorhinal cortex, while the other hallmark of AD, β-amyloid deposition, begins to accumulate in the neocortex. From our data showing such a strong correlation, we may hypothesize that altered methylation within *CRTC1* may be part of the molecular signature of p-tau pathology in the hippocampus. Anyhow, the relationship between *CRTC1* gene and p-tau pathology merits further investigations.

We also found a mild but otherwise significant decrease in *CRTC1* mRNA expression in AD hippocampus compared to controls (Fig. 2). This finding is in concordance with a previous report that showed both total and phosphorylated CRTC1 protein downregulation in human hippocampus affected by AD [19]. Moreover, *CRTC1* mRNA expression has been found to be decreased in other neurodegenerative conditions and brain diseases, such as frontotemporal lobar degeneration with progranulin mutations [34] and Down syndrome [35] (Additional file 1: Figure S5). Down syndrome is considered a natural model for AD, since most people affected by Down syndrome will develop early AD [36]. Interestingly, a significant decrease in *CRTC1* mRNA levels was shown for *CRTC1* transcript variant 1 (NM_015321) but not for *CRTC1* transcript variant 3 (NM_001098482) in Down syndrome adult brain [35] (Additional file 1: Figure S5), which is in line with our present work.

To understand better the relationship between methylation and expression in our study we tested the correlation between *CRTC1* methylation and mRNA levels. Among control hippocampus samples, we found an inverse relationship between methylation and expression at the Prom1 region. In other words, lower levels of DNA methylation within Prom1 showed a trend to be correlated with higher levels of *CRTC1* mRNA (r_Spearman_ = −0.949, *p* = 0.051). This correlation is consistent with the classical model of epigenetic regulation where loss of methylation in the promoter region results in an increase in gene expression, indicating that *CRTC1* is probably regulated by DNA methylation under normal circumstances. However, no correlation between methylation and expression was identified among AD patients in our analysis, suggesting that proper functioning of DNA methylation in regulating *CRTC1* expression may be disturbed in the AD context.

Finally, we show significantly decreased mRNA levels of several *CRTC1*-dependent genes, such as *BDNF*, *EGR4*, and *EGR1* in AD hippocampus compared to control hippocampus (*p*-value < 0.05) (Additional file 1: Figure S4), which adds consistency to the present study. These results are in accordance with previous reports performed on AD in vitro models, murine models and human brain. For instance, oligomeric Aβ peptide downregulates *BDNF* levels via Aβ-induced CREB transcriptional downregulation in human neuroblastoma (SH-SY5Y) cells [34]. In murine models, it has been largely demonstrated that several *CRTC1*-dependent genes are downregulated in the brain tissue affected by AD pathology, including *BDNF* and *EGR4* [17, 18, 35]. Most interestingly, not only are *BDNF* mRNA levels decreased in AD human frontal cortex but also *BDNF* mRNA levels correlate with cognitive performance of patients, assessed by Mini-Mental State Examination scores, as was shown in a recent report [36].

On the other hand, c-fos mRNA expression was significantly increased in AD cases compared to controls in our set of samples (*p*-value < 0.005) (Additional file 1: Figure S4). Remarkably, this is in good agreement with the results obtained from earlier studies that used AD human hippocampus to assess c-fos expression [37-39] but, in turn, differs from several reports that have shown c-fos mRNA levels to be decreased in AD murine models [17, 18, 35]. This discrepancy among the results obtained from murine and human studies regarding c-fos mRNA expression in the context of AD pathology will need further research.

## Conclusions

In conclusion, this study provides evidence that *CRTC1* methylation levels are altered in the human hippocampus affected by AD. It also suggests that the functional relationship between DNA methylation and gene expression for *CRTC1* may be altered in the AD context. Further studies are needed to better understand the precise role that *CRTC1* methylation plays in AD pathophysiology.

## Additional files

Additional file 1: Figure S1. Pictures obtained at 10x from the most representative cases showing different degrees of protein deposit. **Figure S2.** Beta-amyloid and tau protein measurement in hippocampal sections. **Figure S3.** Maps of the two CRTC1 regions analyzed by bisulfite sequencing cloning in human hippocampus. **Figure S4.** mRNA expression levels of CRTC1 downstream genes in human AD hippocampus compared to controls. **Figure S5.** Gene Expression Omnibus (GEO) data analysis for CRTC1 mRNA expression levels in Down syndrome and Frontotemporal Lobar Degeneration with progranulin mutations. (PDF 974 kb) Additional file 2: Supplemental Tables. **Table S1.** Brain sample set characteristics. **Table S2.** Bisulfite and quantitative-PCR primers. (PDF 308 kb)

## Abbreviations

AD: Alzheimer’s disease
APP: amyloid precursor protein
APS: amyloid plaque score
*BDNF*: Brain-derived Neurotrophic Factor
bp: base pair
cDNA: complementary DNA
ChIP-seq: chromatin immunoprecipitation sequencing
CpG: cytosine-phosphate-guanine dinucleotide
*CREB*: cAMP-responsive element binding
*CRTC1*: *CREB* regulated transcription coactivator 1
*EGR1*: Early growth response protein 1
*EGR4*: Early growth response protein 4
ENCODE: Encyclopedia of DNA Elements
GEO: Gene Expression Omnibus
HMM: Hidden Markov Model
mRNA: messenger RNA
PCR: polymerase chain reaction
Prom 1: promoter number 1
Prom2: promoter number 2
p-tau: hyperphosphorylated tau
RT-qPCR: real time-qPCR
UCSC: University of California, Santa Cruz

## References

[1] Conkright MD, Canettieri G, Screaton R, Guzman E, Miraglia L, Hogenesch JB (2003). TORCs: transducers of regulated CREB activity. Mol Cell.

[2] Bourtchuladze R, Frenguelli B, Blendy J, Cioffi D, Schutz G, Silva AJ (1994). Deficient long-term memory in mice with a targeted mutation of the cAMP-responsive element-binding protein. Cell.

[3] Impey S, Smith DM, Obrietan K, Donahue R, Wade C, Storm DR (1998). Stimulation of cAMP response element (CRE)-mediated transcription during contextual learning. Nat Neurosci.

[4] Guzowski JF, McGaugh JL (1997). Antisense oligodeoxynucleotide-mediated disruption of hippocampal cAMP response element binding protein levels impairs consolidation of memory for water maze training. Proc Natl Acad Sci U S A.

[5] Benito E, Valor LM, Jimenez-Minchan M, Huber W, Barco A (2011). cAMP response element-binding protein is a primary hub of activity-driven neuronal gene expression. J Neurosci.

[6] Kovacs KA, Steullet P, Steinmann M, Do KQ, Magistretti PJ, Halfon O (2007). TORC1 is a calcium- and cAMP-sensitive coincidence detector involved in hippocampal long-term synaptic plasticity. Proc Natl Acad Sci U S A.

[7] Sekeres MJ, Mercaldo V, Richards B, Sargin D, Mahadevan V, Woodin MA (2012). Increasing CRTC1 function in the dentate gyrus during memory formation or reactivation increases memory strength without compromising memory quality. J Neurosci.

[8] Kang MG, Byun K, Kim JH, Park NH, Heinsen H, Ravid R (2015). Proteogenomics of the human hippocampus: the road ahead. Biochim Biophys Acta.

[9] Watts AG, Sanchez-Watts G, Liu Y, Aguilera G (2011). The distribution of messenger RNAs encoding the three isoforms of the transducer of regulated cAMP responsive element binding protein activity in the rat forebrain. J Neuroendocrinol.

[10] Zhou Y, Wu H, Li S, Chen Q, Cheng XW, Zheng J (2006). Requirement of TORC1 for late-phase long-term potentiation in the hippocampus. PLoS One..

[11] Ch'ng TH, Uzgil B, Lin P, Avliyakulov NK, O'Dell TJ, Martin KC (2012). Activity-dependent transport of the transcriptional coactivator CRTC1 from synapse to nucleus. Cell.

[12] Li S, Zhang C, Takemori H, Zhou Y, Xiong ZQ (2009). TORC1 regulates activity-dependent CREB-target gene transcription and dendritic growth of developing cortical neurons. J Neurosci.

[13] Breuillaud L, Rossetti C, Meylan EM, Merinat C, Halfon O, Magistretti PJ (2012). Deletion of CREB-regulated transcription coactivator 1 induces pathological aggression, depression-related behaviors, and neuroplasticity genes dysregulation in mice. Biol Psychiatry.

[14] Robida-Stubbs S, Glover-Cutter K, Lamming DW, Mizunuma M, Narasimhan SD, Neumann-Haefelin E (2012). TOR signaling and rapamycin influence longevity by regulating SKN-1/Nrf and DAF-16/FoxO. Cell Metab.

[15] Burkewitz K, Morantte I, Weir HJ, Yeo R, Zhang Y, Huynh FK (2015). Neuronal CRTC-1 governs systemic mitochondrial metabolism and lifespan via a catecholamine signal. Cell.

[16] Xue ZC, Wang C, Wang QW, Zhang JF (2015). CREB-regulated transcription coactivator 1: important roles in neurodegenerative disorders. Sheng Li Xue Bao.

[17] Espana J, Valero J, Minano-Molina AJ, Masgrau R, Martin E, Guardia-Laguarta C (2010). beta-Amyloid disrupts activity-dependent gene transcription required for memory through the CREB coactivator CRTC1. J Neurosci.

[18] Saura CA (2012). CREB-regulated transcription coactivator 1-dependent transcription in Alzheimer's disease mice. Neurodegener Dis.

[19] Parra-Damas A, Valero J, Chen M, Espana J, Martin E, Ferrer I (2014). Crtc1 activates a transcriptional program deregulated at early Alzheimer's disease-related stages. J Neurosci.

[20] Heyn H, Li N, Ferreira HJ, Moran S, Pisano DG, Gomez A (2012). Distinct DNA methylomes of newborns and centenarians. Proc Natl Acad Sci U S A.

[21] Horvath S, Zhang Y, Langfelder P, Kahn RS, Boks MP, van Eijk K (2012). Aging effects on DNA methylation modules in human brain and blood tissue. Genome Biol.

[22] Weidner CI, Lin Q, Koch CM, Eisele L, Beier F, Ziegler P (2014). Aging of blood can be tracked by DNA methylation changes at just three CpG sites. Genome Biol.

[23] Hernandez DG, Nalls MA, Gibbs JR, Arepalli S, van der Brug M, Chong S (2011). Distinct DNA methylation changes highly correlated with chronological age in the human brain. Hum Mol Genet.

[24] Heyn H, Esteller M (2012). DNA methylation profiling in the clinic: applications and challenges. Nat Rev Genet.

[25] Bergman Y, Cedar H (2013). DNA methylation dynamics in health and disease. Nat Struct Mol Biol.

[26] Montine TJ, Phelps CH, Beach TG, Bigio EH, Cairns NJ, Dickson DW (2012). National Institute on Aging-Alzheimer's Association guidelines for the neuropathologic assessment of Alzheimer's disease: a practical approach. Acta Neuropathol.

[27] Bell JE, Alafuzoff I, Al-Sarraj S, Arzberger T, Bogdanovic N, Budka H (2008). Management of a twenty-first century brain bank: experience in the BrainNet Europe consortium. Acta Neuropathol.

[28] Schneider CA, Rasband WS, Eliceiri KW (2012). NIH Image to ImageJ: 25 years of image analysis. Nat Methods.

[29] Miller SA, Dykes DD, Polesky HF (1988). A simple salting out procedure for extracting DNA from human nucleated cells. Nucleic Acids Res.

[30] Li LC, Dahiya R (2002). MethPrimer: designing primers for methylation PCRs. Bioinformatics.

[31] Kumaki Y, Oda M, Okano M (2008). QUMA: quantification tool for methylation analysis. Nucleic Acids Res..

[32] Livak KJ, Schmittgen TD (2001). Analysis of relative gene expression data using real-time quantitative PCR and the 2(-Delta Delta C(T)) Method. Methods.

[33] Kent WJ, Sugnet CW, Furey TS, Roskin KM, Pringle TH, Zahler AM (2002). The human genome browser at UCSC. Genome Res.

[34] Chen-Plotkin AS, Geser F, Plotkin JB, Clark CM, Kwong LK, Yuan W (2008). Variations in the progranulin gene affect global gene expression in frontotemporal lobar degeneration. Hum Mol Genet.

[35] Lockstone HE, Harris LW, Swatton JE, Wayland MT, Holland AJ, Bahn S (2007). Gene expression profiling in the adult Down syndrome brain. Genomics.

[36] Hartley D, Blumenthal T, Carrillo M, DiPaolo G, Esralew L, Gardiner K (2015). Down syndrome and Alzheimer's disease: common pathways, common goals. Alzheimers Dement.

