# Supplementary material for: CRTC1 gene is differentially methylated in the human hippocampus in Alzheimer’s disease
Source: Alzheimers Res Ther. 2016 Apr 19;8:15. doi: 10.1186/s13195-016-0183-0 (PMC4837517; doi:10.1186/s13195-016-0183-0)
Supplement: Additional file 1: Figure S1. — Pictures obtained at 10x from the most representative cases showing different degrees of protein deposit. Figure S2. Beta-amyloid and tau protein measurement in hippocampal sections. Figure S3. Maps of the two CRTC1 regions analyzed by bisulfite sequencing cloning in human hippocampus. Figure S4. mRNA expression levels of CRTC1 downstream genes in human AD hippocampus compared to controls. Figure S5. Gene Expression Omnibus (GEO) data analysis for CRTC1 mRNA expression levels in Down syndrome and Frontotemporal Lobar Degeneration with progranulin mutations. (PDF 974 kb) [file 13195_2016_183_MOESM1_ESM.pdf]

Supplemental Figures

Supplemental Figure S1

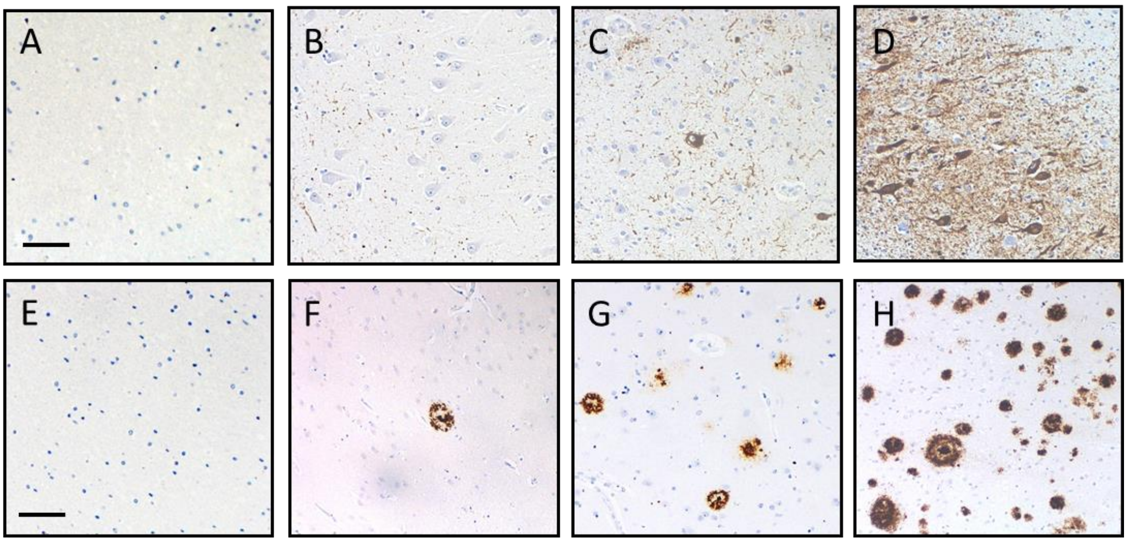

Pictures obtained at 10 x from the most representative cases showing different degree of protein deposit. A-D: p-Tau stain. Density degree of neurophil threads and tangles (A, control; B, mild; C, moderate; D, severe). **E-H**: Amyloid stain. Density degree of neuritic plaques (E, control; F, mild; G, moderate; H,severe). Scale bar: 200 µm

Supplemental Figure S2

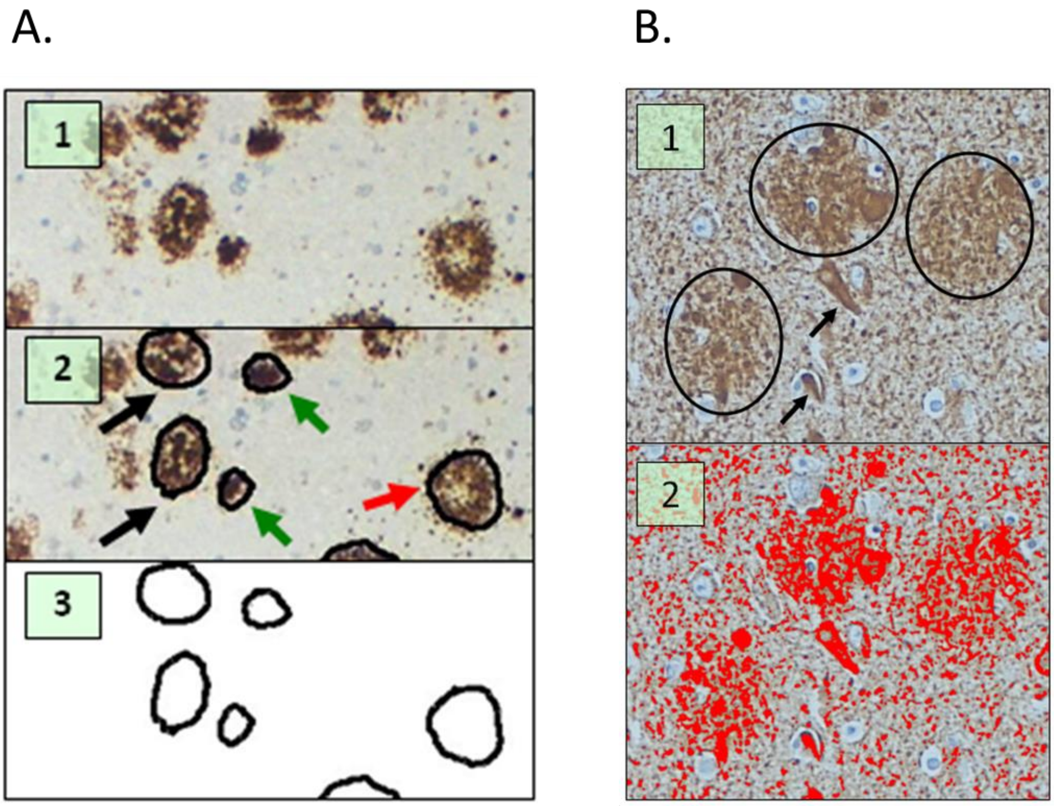

Beta-amyloid and tau protein measurement in hippocampal sections. A. The figure illustrates the method to measure amyloid deposits by using the ImageJ software. A1. Original figure of amyloid immunostained section obtained at 10x. A2. Selection of the different focal deposits patterns: neuritic plaques (black arrows), compact plaques (green arrows) and immature plaques (red arrow). A3. Analysis of the selection by using the ImageJ software. B. Tau deposits measurement. B1. Tau immunostained section obtained at 10 x. The figure shows different deposits of tau protein: Neurofibrillary tangles (black arrows), neuritic plaques (black circles) and neuropil threads (background). B2: Selection of the whole deposit with the ImageJ software.

Supplemental Figure S3

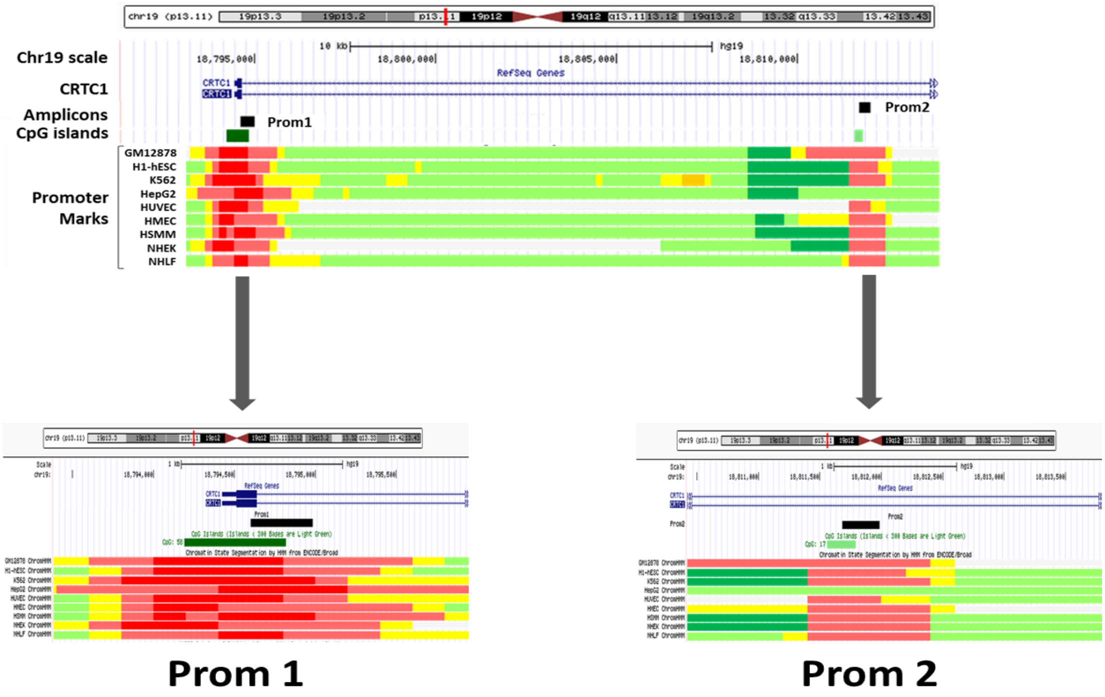

Maps of the two *CRTC1* regions analyzed by bisulfite sequencing cloning in human hippocampus. The figure shows zoom-out mapping of the two amplicons (Prom1 and Prom2) within the two promoter regions of the *CRTC1* gene that were examined by bisulfite cloning sequencing. *CRTC1* is located on the short arm of chromosome 19 (chr19:18,794,425-18,893,143-GRchr17/hg19 coordinates). At the bottom of each graph, predicted functional elements are shown for each of nine human cell lines explored by Chromatin immunoprecipitation (ChIP) combined with massively parallel DNA sequencing. Boxes represent promoter regions (red), enhancers (yellow), transcriptional transition & elongation (dark green) and weak transcribed regions (light green). CpG islands are also represented by isolated green boxes. The track was obtained from *Chromatin State Segmentation by HMM from ENCODE/Broad track* shown at the UCSC Genome Browser.

Supplemental Figure S4

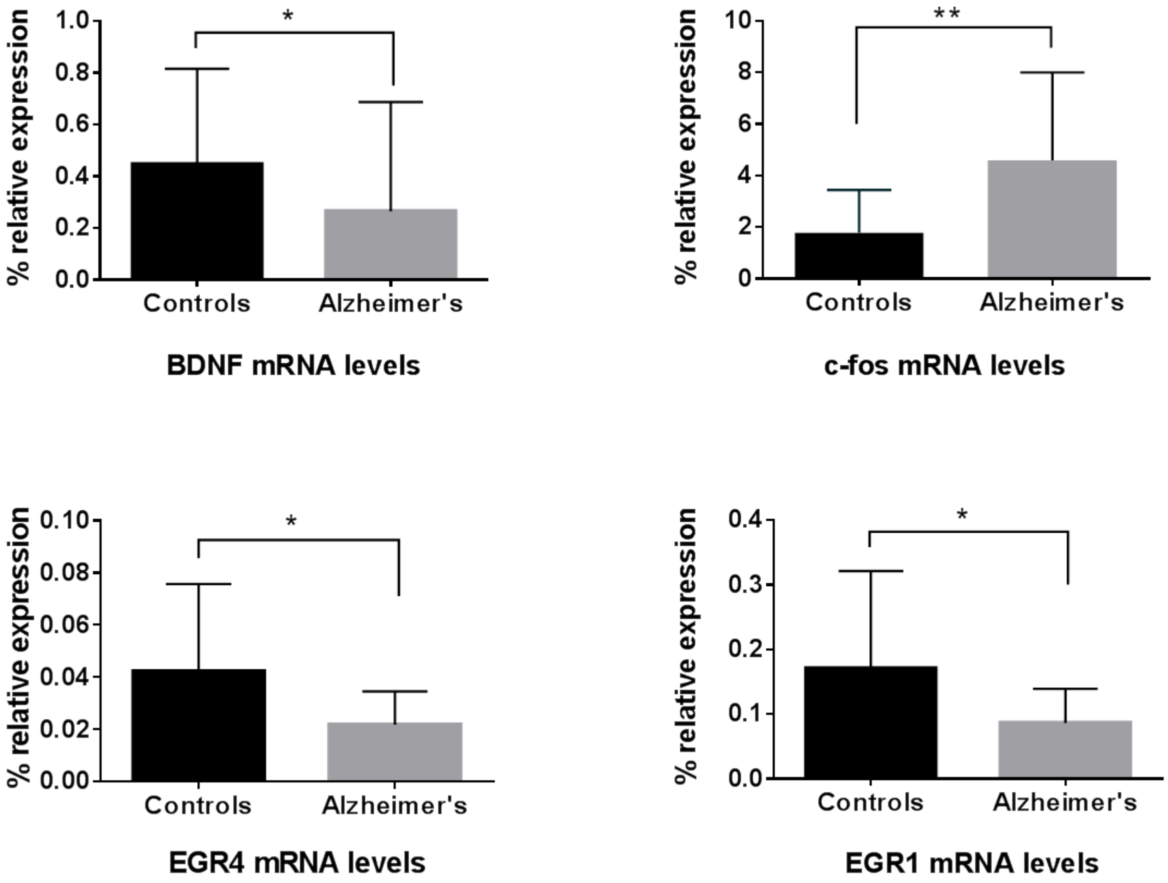

mRNA expression levels of CRTC1 downstream genes in human AD hippocampus compared to controls. The graphs show a significant decrease in BDNF, EGR4, and EGR1 mRNA levels in AD hippocampal samples compared to control hippocampal samples. On the contrary, c-fos mRNA levels were found to be significantly increased in the AD hippocampus compared to controls. Boxes represent percentage of mRNA expression relative to the geometric mean of GAPDH and ACTB housekeeping genes expression. Bars represent the standard error of the mean.*p-value<0.05; ** p-value<0.005.

Supplemental Figure S5

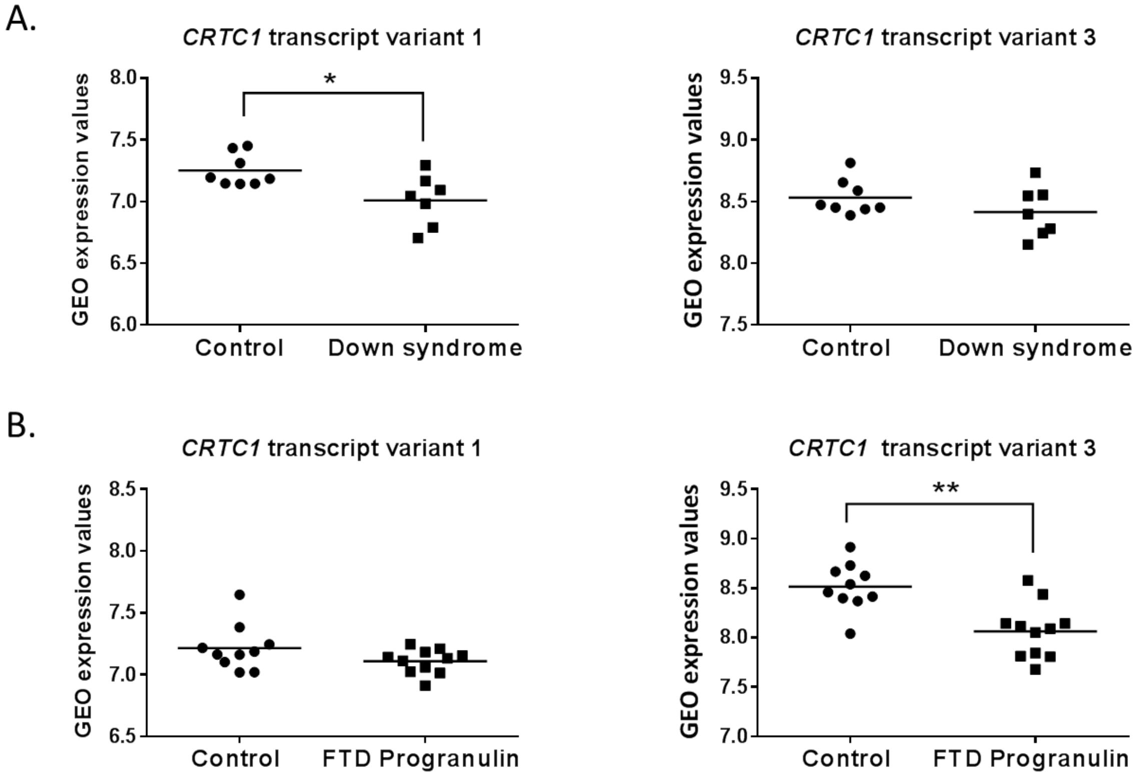

Gene Expression Omnibus (GEO) data analysis for *CRTC1* mRNA expression levels in Down syndrome and Frontotemporal Lobar Degeneration with progranulin mutations. Graphs represent *CRTC1* mRNA expression levels obtained from the GEO database for the Affymetrix Human Genome U133A Array performed on postmortem human brain in different pathological conditions, such as Down syndrome (A) and Frontotemporal Lobar Degeneration with Progranulin gene mutations (B). Separate expression levels for both transcripts, *CRTC1* transcript variant 1 (Affymetrix ID probe 207159_x_at) and transcript variant 3 (Affymetrix ID probe 213091_at) are shown on the left and right side respectively. *p-value<0.05; ** p-value<0.01
