# Supplementary material for: CRTC1 gene is differentially methylated in the human hippocampus in Alzheimer’s disease
Source: Alzheimers Res Ther. 2016 Apr 19;8:15. doi: 10.1186/s13195-016-0183-0 (PMC4837517; doi:10.1186/s13195-016-0183-0)
Supplement: Additional file 2: — Supplemental Tables. Table S1. Brain sample set characteristics. Table S2. Bisulfite and quantitative-PCR primers. (PDF 308 kb) [file 13195_2016_183_MOESM2_ESM.pdf]

Supplemental Tables

Supplemental Table S1. Brain sample set characteristics.

No.	Diagnosis	ABC score	APS	Age at death (years)	Gender	PMI (h)	Region hippocampus
1	Control	control	NA	61	male	8	yes
2	Control	control	NA	81	male	10.5	yes
3	Control	control	NA	43	female	3	yes
4	Control	control	NA	88	male	9	yes
5	Control	control	NA	53	male	7	yes
6	Control	control	NA	41	male	3.5	yes
7	Control	control	NA	28	male	6	yes
8	Control	control	NA	46	female	7	yes
9	Control	control	NA	69	male	12	yes
10	Control	control	NA	19	female	NA	yes
11	Control	control	NA	26	male	6.2	yes
12	Control	control	NA	54	male	18	yes
13	AD	A1B1C1	0.00	60	male	15.3	yes
14	AD	A1B1C1	2.00	85	male	3.2	yes
15	AD	A1B1C1	0.00	66	female	1.4	yes
16	AD	A1B1C1	0	85	female	4.3	yes
17	AD	A1B2C1	0.00	88	female	33	yes
18	AD	A1B2C1	3.00	96	female	1.5	yes
19	AD	A1B2C2	0.33	79	female	13	yes
20	AD	A1B2C3	2.33	84	female	13	yes
21	AD	A2B2C3	2.00	98	female	23	yes
22	AD	A3B2C3	2.67	85	female	NA	yes
23	AD	A3B2C3	3.67	83	male	9	yes
24	AD	A3B2C3	6.67	69	female	4.3	yes
25	AD	A1B2C2	1.00	81	female	9	yes
26	AD	A3B2C3	6.67	98	female	3	yes
27	AD	A2B2C2	5.00	88	male	3.5	yes
28	AD	A2B2C2	2.33	91	female	10	yes
29	AD	A2B2C2	1.33	84	male	3.3	yes
30	AD	A2B2C2	3.00	97	female	NA	yes
31	AD	A2B2C3	1.33	78	male	5	yes
32	AD	A3B2C1	1.33	90	female	3	yes
33	AD	A3B3C2	3.00	92	female	14	yes
34	AD	A3B3C3	4.00	77	female	11	yes
35	AD	A3B3C2	7.00	82	female	9	yes
36	AD	A3B3C2	8.00	91	male	5	yes
37	AD	A2B3C3	5.67	77	female	4	yes
38	AD	A3B3C3	3.33	93	female	3	yes
39	AD	A3B3C3	8.00	86	female	2.3	yes

40	AD	A3B3C3	4.33	61	male	10	yes
41	AD	A3B3C3	9.67	70	male	2.35	yes
42	AD	A3B3C3	8.33	59	male	4	yes

The table shows the characteristic of the samples included in the study. No.: Number; ABC score: NPD: no protein deposit; APS: amyloid plaque score; h: hours; AD: Alzheimer's disease; PMI: post mortem interval.

Supplemental Table S2. Bisulfite and quantitative-PCR primers.

ID	Accesion number	PCR Purpose	Amplicon size	Tm	Forward Primer	Tm2	Reverse Primer
CRTC1 Prom1	NA	Bisulfite PCR	386 bp	59.93	GGGTAATAATGATGTAGGGATATTTGTT	55.09	AAAATCATAAAAAACCTAAACCTAAC
CRTC1 Prom2	NA	Bisulfite PCR	302 bp	57.64	GATTGAAAGTAGAGGTTTTTTGTTG	58.52	TAAACCTTCAAACTCCTCCTTCTAC
CRTC1_qPCR1	NM_015321; NM_001098482	qPCR	149 bp	62.6	AGATGGCGACTTCGAACAATC	61.8	CTGGGATTTCTGGAGCTGG
CRTC1_qPCR2	NM_015321; NM_001098482	qPCR	136 bp	62.4	CATCCCCAACATCATCCTCAC	61.7	GATCTTGAGTTCGTCCAGGG
CRTC1_qPCR3	NM_001098482	qPCR	128 bp	62	CAGCTCCAGAAATCCCAGTAC	61.7	AGAAATCCGCTGGGCTG
BDNF_q	NM_170733	qPCR	147 bp	61.7	GCTGCCTTGATGGTTACTTTG	62.4	TGTCCTCGGATGTTTGCTTC
cfos_q	NM_005252	qPCR	117 bp	61.8	TTGTGAAGACCATGACAGGAG	62.6	CCATCTTATTCCTTTCCCTTCGG
ERG4_q	NM_001965	qPCR	150 bp	62	AGTTTTCCGAACCCGACG	62.9	TGTTCAAAGCCCAGCTCAAG
EGR1 _q	NM_001964	qPCR	135 bp	62.2	CAGCACCTTCAACCCTCAG	61.7	AGTCGAGTGGTTTGGCTG

The table shows the primer pairs used in the study. Amplified transcripts are identified by RefSeq Accession or GeneBank accesion number. ID: identification; qPCR: quantitative PCR; bp: base pair; Tm: Melting Temperature.
